# Forward-backward translation and content validity study of the German version of the Patient-Reported Apnea Questionnaire

**DOI:** 10.1186/s41687-026-01246-3

**Published:** 2026-11-05

**Authors:** Alina Wildenauer, Christoph Schöbel, Pia Awater, Fenneke Attema, Mariele Stockhoff, Inger Abma, Marcel Braun

**Affiliations:** 1 University Clinic Essen, West German Lung Centre, Ruhrlandklinik - Centre for Sleep and Telemedicine, Essen, Germany; 2 Institute for German and Dutch Studies, University of Duisburg-Essen, Essen, Germany; 3 IQ Health Science Department, Radboud University Medical Center, Nijmegen, Netherlands; 4 Independent Researcher, Eindhoven, The Netherlands

**Keywords:** Health-related quality of life, Obstructive sleep apnea, Forward-backward translation, Content validity study, Patient-reported outcome measures

## Abstract

**Background:**

Health-related quality of life (HRQOL) assessment is an important component of chronic disease management, providing insights beyond clinical parameters. In obstructive sleep apnea (OSA), no comprehensive German-language patient-reported outcome measure (PROM) is currently available for routine clinical use. This study aims to adapt the Patient-Reported Apnea Questionnaire (PRAQ), a validated OSA-specific HRQOL instrument, for German-speaking patients.

**Methodology:**

The German adaptation of the PRAQ followed a forward-backward translation process involving three translators. After harmonization, content validity was assessed through cognitive interviews with 20 OSA patients and an online survey among 14 sleep medicine experts. Feedback from both groups was incorporated into the final version of the questionnaire.

**Results:**

The translation and adaptation process was conducted between April 2025 and March 2026. Two forward translations, one back-translation, and a final harmonization were performed. Patients identified issues mainly related to item relevance, conceptual overlap and response formats in the domains of daily activity, emotions and social interactions. Experts confirmed overall good comprehensibility but highlighted some concerns regarding item relevance and response scales. Based on patient and expert feedback, eight modifications were implemented, including clarifications of OSA-related terms, response options, and specific item wording, resulting in the final German PRAQ version.

**Conclusion:**

The German version of the PRAQ demonstrates decent content validity and comprehensibility, while reflecting the heterogeneous experiences of patients with OSA. Although some items were considered less relevant by certain patients, no essential domains were identified as missing. The study was approved by the ethics committee of the ​ University of Duisburg-Essen (25-12560-BO), and all participants provided written informed consent.

**Supplementary Information:**

The online version contains supplementary material available at https://doi.org/10.1186/s41687-026-01246-3.

## Introduction

The assessment of health-related quality of life (HRQOL) in chronic illnesses is an essential component of modern healthcare, as it systematically incorporates the patient’s perspective into clinical care and facilitates disease-specific management beyond biomarkers and isolated clinical parameters [1]. This is typically done using patient-reported outcome measures (PROMs), which are usually questionnaires completed by patients themselves. While generic HRQOL instruments allow comparisons across different diseases and populations, disease-specific HRQOL instruments capture dimensions particularly relevant to a given condition, including social, emotional, and functional impairments in addition to symptom burden [2]. Obstructive Sleep Apnea (OSA) is among the most prevalent sleep-related disorders, characterized by a partial or total collapse of the upper airway soft tissues, resulting in nocturnal arousals and oxygen desaturation [3]. Cardiovascular diseases, such as increased risk of myocardial infarction and stroke, are associated with OSA [4], while excessive daytime sleepiness, snoring, cognitive impairment, lower quality of life and increased risk of accidents are common symptoms [5, 6]. A variety of disease-specific HRQOL instruments for OSA are available, ranging from brief questionnaires requiring approximately three minutes to complete and focussing on few specific quality of life (QOL) domains [7] to exhaustive tools with up to 84 items [8] and even visual scales [9]. At present, a comprehensive German-language PROM for assessing HRQOL in OSA, specifically intended for routine clinical implementation with good measurement properties, does not exist. HRQOL PROMs established in clinical practice in Germany are the Functional Outcome of Sleep Questionnaire (FOSQ), and its short version the FOSQ-10 [10], which focuses solely on the impact of sleepiness on daily functioning, while other PROMs are used for screening or diagnostics. Two systematic reviews from 2017 [11] and 2026 [12] assessed the quality of HRQOL instruments for OSA, resulting in five instruments deemed of at least moderate psychometric quality and good content validity according to the COnsensus based Standards for the selection of health Measurement Instruments (COSMIN). The Quebec Sleep Questionnaire (QSQ) [13], Maugeri Obstructive Sleep Apnea (MOSAS) [14], Calgary Apnea Quality of Life Index(SAQLI) [8], Obstructive Sleep Apnea Patient-Oriented Severity Index (OSAPOSI) [15] and the Patient-Reported Apnea Questionnaire (PRAQ) [16], thus hold the highest level of recommendation. The PRAQ is an originally Dutch PROM comprising 40 items across 10 domains (*symptoms at night*,* sleepiness*,* tiredness*,* daily activities*,* unsafe situations*,* memory and concentration*,* quality of sleep*,* emotions*,* social and health concerns*). The PRAQ was selected for translation because it covers a broad range of OSA-related symptoms and their emotional, functional, and social impact besides important clinical symptoms such as sleepiness and symptoms at night, while being specifically designed for routine clinical practice, with a completion time of approximately 15 min. Its items are derived from the QSQ, MOSAS, and SAQLI, combining content from existing high quality PROMs into a composite instrument with good content validity and sound psychometric properties. This study represents the first step towards making this broader, patient-centred assessment available to German-speaking patients, with further evaluation of the adapted version’s measurement properties required.

## Methods

We used a forward-backward design for the translation process with three translators in total. After the harmonization of the questionnaire, a content validity study with 20 OSA patients in form of cognitive interviews and an online survey study with 14 sleep research and sleep medicine experts was conducted, followed by finalization of the questionnaire.

This study was approved by the ethics committee of the University of Duisburg-Essen (25-12560-BO)​ and written informed consent was obtained from all participants.

### Translation process

We obtained permission from the instrument developer to translate the questionnaire and evaluate its measurement properties, received the original Dutch version of the PROM, and discussed the final translation and the analysis of the cognitive interviews with the developer. We closely followed the recommended steps of a forward-backward translation wherever possible [17, 18]. Two independent translators, MS, a native German speaker and former clinical success specialist in sleep science working in the Netherlands, and PA, a native German speaker and research assistant at the Institute for German and Dutch Studies at the University of Duisburg-Essen, translated the PROM from Dutch into German. Both translators were instructed to keep the translation as close to the original wording as possible while paying particular attention to culturally sensitive terms and expressions. The guideline by Brislin et al. [19] was provided as a general framework. MS prepared a forward translation using an AI‑assisted approach (ChatGPT, version: GPT-4o), whereas PA produced an independent forward translation without digital aids, relying solely on dictionaries. MS and PA required two weeks and four weeks, respectively, completing the full translation. Subsequently, the German translation was harmonized by AW and PA by discussing and resolving discrepancies in wording and sentence structure. The harmonized German version was then provided to FA, a native Dutch speaker with a bachelor’s degree in German language and culture from Utrecht University and current master’s student for German and Dutch language at the University of Duisburg-Essen, for back-translation without prior access to the original version. FA took two weeks to complete the translation, which was done without digital tools. During the final harmonization meeting, AW, FA, and PA reviewed the original version, the harmonized German version, and the back-translation side by side. For the final revision of the PROM, AW and MB proofread the version and discussed necessary changes based on the analysis of patients’ and experts ‘opinions which were reviewed by IA.

### Cognitive interviews with patients

Cognitive interviews with OSA patients were conducted using think‑aloud and probing techniques [20] in either a face-to-face or video-based setting, which is also recommended by the COSMIN standard [21]. This method was used to encourage patients to express their thoughts freely, without the constraints of predefined questions or rating scales. This allowed nuanced feedback to emerge and enabled targeted probing when clarification was needed, including regarding item relevance. All interviews were conducted by AW and all 20 patients were recruited via an OSA patient organization via email invitation. Two rounds of cognitive interviews were conducted with 10 patients each in line with Beatty and Willis [20], who mention that data saturation may be achieved with 5–15 interviews through an iterative process. We included all patients that agreed to participate. At the beginning of each interview, participants received a brief explanation of the study context, namely that the interview was part of an evaluation of a questionnaire translated into German. Due to data protection requirements, no audio recordings or verbatim transcripts were collected or analysed, rather, patients’ opinions were paraphrased and important quotes were written down verbatim. Participants were encouraged to provide spontaneous and free responses and were informed that there were no “right” or “wrong” answers. They were also informed that follow-up questions would be asked when clarification was needed. They were asked to talk about their current situation and the time before initiating of an OSA therapy. If patients had trouble coming up with an answer, the answer was too short or they raised an interesting topic that warranted further inquire, probing questions were used, for example by assessing conceptual understanding (e.g., “What does this term mean to you?”) or requesting paraphrasing (e.g., “Can you restate this in your own words?”). In addition, the recall period and the appropriateness of the response categories were evaluated. Cognitive interviews were conducted in two rounds. After the first 10 interviews, preliminary revisions addressing frequently raised issues were implemented. In the second round, participants were first presented with the revised version; the original version was subsequently shown for comparison and discussion. To facilitate the analysis of qualitative data, items were classified as problematic if ≥ 30% of patients (*n* = 6) provided critical comments on relevance or comprehensibility.

### Online survey with sleep experts

Experts in sleep science and sleep medicine were recruited via email and invited to complete an online survey administered in LimeSurvey (www.limesurvey.org). Responses were collected anonymously and participants accessed the survey via individualized tokens. The experts were included through convenience sampling, representing the total number of experts recruited who provided responses, while 24 were invited initially. The experts were asked to evaluate the items’ relevance, completeness, and comprehensibility. Relevance and comprehensibility were rated on a 4‑point scale (1 = very relevant/very clear; 2 = relevant/clear; 3 = partly relevant/clear; 4 = not relevant/not clear) [22]. For each domain, experts could additionally indicate missing content. The appropriateness of the response categories was also assessed, and free‑text fields were provided for suggestions and proposed revisions. Content validity was quantified using the Content Validity Index (CVI) [22] at the item (I‑CVI), domain (D‑CVI), and scale levels (S‑CVI). For each item, ratings of 1 (“very relevant/very clear”) and 2 (“relevant/clear”) were dichotomized into endorsement and summed across experts. The sum of these ratings divided by the number of experts yielded the I‑CVI. Domain-level CVI (D‑CVI) was calculated as the mean of I‑CVI values within each domain, and the scale-level CVI (S‑CVI) as the mean of I‑CVI values across all items. Although this method is primarily intended to assess relevance (REL‑CVI), comprehensibility CVI (COM‑CVI) was calculated in the same fashion. Acceptability thresholds were predefined according to existing standards [22] as I‑CVI ≥ 0.78 and S‑CVI ≥ 0.90.

## Results

The entire process, from translation through final harmonization and expert survey, was conducted between April 2025 and March 2026.

### Forward translation

In total, *N* = 54 differences were identified between the two translations (recurrent differences, e.g., in domain introductions, were counted as a single difference). The majority of these differences concerned wording or synonyms. Some differences were semantic in nature and required further discussion, incorporating consideration of connotation and cultural appropriateness. In addition, one version more often used concise nominalizations, whereas the other tended to employ verb-based paraphrases resulting in longer sentence structures overall. Differences in tense constructions were also common, yet the original version is a mixed-tense questionnaire with a strong present perfect dominance, so we tried to stay faithful to the original version, if possible. We tended to select the shorter wording when the meaning did not differ between versions and made case-by-case decisions on specific terms, considering closeness to the original and cultural appropriateness in German. See Table 1 for selected examples.

### Backward translation and final harmonization

Differences between the original and the back-translated version were again, mainly due to variation of specific wordings. Similarly, some wording decisions regarding semantics, usually in the same section as discussed within the forward translation process, occurred. Comparison of the three versions further showed that the original and the back-translation mainly used infinitive constructions, whereas the German translation more frequently employed nominalizations, which are more conventional and concise in German. Differences were also observed in tense usage. Both translators agreed that tense selection between the simple past and the present perfect is relatively flexible in both German and Dutch language. Consistent with the original, the simple past was preferred because it generally results in more concise sentence structures, thus during final harmonization, systematic discrepancies were resolved to ensure consistent tense and sentence structure, while remaining wording decisions were made on a case-by-case basis to produce a culturally appropriate instrument that remains as close as possible to the original. See Table 1 for selected examples.

**Table 1:** Selected examples from the translation process

Category	Example German/Dutch		English Equivalent*		Choice with reasoning
**Forward Translation**					
Synonyms and Wording	“letzten” vs. “vergangenen” in domain description		„past“ vs. „last“		choosing „letzten“ due to space constraints, as no substantial differences in comprehension or interpretation were expected
Semantics	„Mühe“ vs. „Anstrengung“ in domain „Daily Activities”		„difficulty“		choosing “Mühe” because it has a slightly more neutral tone in German, whereas “Anstrengung” can carry a more negative connotation
	“genervt” vs. “unwohl gefühlt” in the domain of “Social Interactions” when asking about other people reacting to ones snoring		feeling “upset” vs. “uneasy”		choosing “unwohl gefühlt” because it more closely reflected the original meaning and was more appropriate for the item
Nominalisations	“nächtliches Erwachen vs. ““aufwachen in der Nacht””		„nocturnal awakening“ vs. “waking up at night”		Choosing mainly nominalisations due to space constraints and common use in German language
Tenses	Simple past (“Hatten Sie”) vs. present perfect (“Haben Sie…gehabt”)		„Did you“ vs. “Had you”		Choosing tense closest to the original and cultural appropriateness in German
**Backward Translation**					
Semantics	“vervelend gevoeld” vs. “ ongemakkelijk” in domain “Social Interaction” when asking about other people reacting to ones snoring		“ feeling “upset” vs. “uneasy”		“ongemakkelijk” and the German “unwohl fühlen” provided a better fit by reflecting social shame and embarrassment.
Synonyms and Wording	“erg” vs. “ernstig” vs. „hevig“ vs. „ontzettend“ vs. “flink” in the domain of “Tiredness”.		“very”		Several alternative synonyms were discussed, but we ultimately selected the German “stark” as the most appropriate, since its common use in other tools and a typical phrase of patients, acknowledging several valid options.

*Where applicable, we chose the wording of the English version provided in the original publication

### Cognitive interviews with patients

In total, *N* = 20 patients participated in two rounds of cognitive interviews from December 2025 to January 2026. The demographics of participants are presented in Table 2. The interviews took on average 60 min (range 30–90 min).

**Table 2:** Characteristics of patient population for cognitive interviews (*N* = 20)

Variable	Categories	Absolute (± SD)	Absolute (%)
Age (years)		69,1 (± 8,14)	
Sex	Male		15 (75%)
	Female		5 (25%)
Marital Status	Married		14 (70%)
	Single		3 (15%)
	Divorced		2 (10%)
	Widowed		1 (5%)
Education Level	Lower secondary school (Hauptschule)		2 (10%)
	Intermediate secondary school (Realschule)		3 (15%)
	Upper secondary school school (Abitur)		2 (10%)
	Vocational training		4 (20%)
	Meister qualification (Meisterbrief)		1 (5%)
	Master’s degree / Diploma		8 (40%)
Employment Status	Full-time		1 (5%)
	Part-time		3 (15%)
	Retired		15 (75%)
	Other (Homemaker)		1 (5%)
Monthly Income (€)	1,000 – < 2,000		2 (10%)
	2,000 – < 3,000		5 (25%)
	3,000 – < 4,000		6 (30%)
	4,000 – < 5,000		1 (5%)
	≥ 5,000		2 (10%)
	No answer		4 (20%)
Time since OSA diagnostic	1 – <2 years		1 (5%)
	2 – <6 years		2 (10%)
	6 – <10 years		5 (25%)
	≥ 10 years		12 (60%)

In the majority of times, patients freely talked about their personal experience regarding the topic raised by an item, yet some items required the frequent use of probing questions, e.g. to differ between “sleepiness” and “tiredness” or to specify how they remembered feeling doing specific tasks. An overview of relevance and comprehensibility of items can be found in Table 3. Relevance concerns were reflected in statements such as “I did not experience this”, “What does this have to do with apnea?”, or “Never had this.” Comprehensibility concerns were indicated by comments such as “What does this mean?”, “In relation to what—only my condition or in general?”, or “This cannot really be answered,” suggesting unclear concepts or wording.

Regarding relevance, 27 items (67.5%) were rated as relevant, whereas 13 items (32.5%) were identified as problematic. Of these, five items (12.5%) were rated as not relevant by approximately three-quarters of respondents: “headache” (Nocturnal Symptoms), “performance” and “household” (Daily Activities), and “short temper” and “strong emotional reaction” (Emotions). Items rated as irrelevant were largely confined to the “Daily Activities” and “Emotions” domains. For “headache,” most participants stated that the symptom does not and did not apply to them (e.g., “that was never a problem for me”). In “Daily Activities”, the first item was typically answered in detail, whereas subsequent items—especially “Energy main activity” and “main activity” and sometimes “performance” were difficult to distinguish between and were often answered similarly to preceding items (e.g., “I already answered this” or “the prior question asks the same thing”). In the domain “Emotions”, “short temper” and “strong emotional reaction” were likewise often perceived as redundant insofar as the item “feeling irritated” covered the aspect of the previous items sufficiently.

In terms of comprehensibility, 34 items (85%) were considered well understood. Comprehensibility closely mirrored relevance, with less comprehensible items being located in the domain of “Daily Activities” and “Emotions”, which participants attributed mainly to insufficient conceptual differentiation between items. The item “relationship problems” in the domain “Social Interaction” asks about whether there was conflict in the relationship in the past four weeks and was repeatedly flagged as problematic, mainly due to unspecific wording (e.g., “what does this have to do with OSA?”) with many suggesting to specify further, e.g. “because of snoring or tiredness”). The recall period was generally considered appropriate, although participants frequently noted that symptoms often persisted beyond the four-week window. Within the first round of cognitive interviews response options were found satisfactory and were well understood, yet almost all participants indicated difficulty with switching response formats within domains; therefore, wording was partially revised to enable a uniform scale. This affected only the domain of “Daily Activities”, where a consistent “never–always” scale was applied in round two. In the domain “Unsafe Situations”, “not applicable” was changed to “I do not drive a car / operate machinery”, for participants often struggled to distinguish “not applicable” from “not at all”. Similarly, in the domain “Social Interaction”, the response option was changed to “I do not have a partnership” for two item due to the same issue. In addition, a brief definition of OSA was added to the introduction (“Sleep apnea means that breathing pauses repeatedly occur during sleep, which disrupts sleep and can have consequences for health”), because some patients raised the point that others might not be familiar with the term, even though experiencing specific symptoms. Furthermore, patients consistently showed difficulties differentiating between sleepiness and tiredness, often defining sleepiness as tiredness and vice versa (“to me, sleepiness is if I just don’t have energy and tiredness is when I could fall asleep everywhere”). All changes were revealed as an alternate option to patients participating in the second round of interviews after the slightly altered PROM was completed, yet the altered version was consistently preferred over the original.

**Table 3:** Overview of relevance and comprehensibility of items rated by patients

Domain	Item No.	Keyword	Not Relevant (%)	Not Comprehensible (%)
Nocturnal Symptoms	1	Snoring	5%	0%
	2	Nocturia	45% *	0%
	3	Choking	25%	0%
	4	Restless sleep	20%	5%
	5	Dry mouth	10%	10%
	6	Headache	85% *	25%
Sleepiness	7	Fighting sleepiness	5%	0%
	8	Falling asleep	5%	10%
	9	Conversation	25%	10%
	10	Watching something	15%	0%
	11	Places	25%	5%
Tiredness	12	Feeling tired	15%	25%
	13	Energy	15%	5%
	14	Waking up tired	25%	0%
Daily Activities	15	Main activity	20%	5%
	16	Energy main activity	40% *	30% *
	17	Performance	70% *	40% *
	18	Hobbies	40% *	10%
	19	Household	75% *	50% *
Unsafe Situations	20	Sleepiness	0%	0%
	21	Traffic concerns	0%	5%
Memory & Concentration	22	Forgetfulness	25%	5%
	23	Concentration	20%	0%
Sleep Quality	24	Falling asleep	35% *	10%
	25	Staying asleep	10%	10%
Emotions	26	Low mood	20%	15%
	27	Anxiety	35% *	15%
	28	Short temper	70% *	45% *
	29	Irritability	20%	15%
	30	Daily life	40% *	5%
	31	Emotional reaction	90% *	60% *
Social Interactions	32	Snoring	5%	5%
	33	Time with family	25%	10%
	34	Guilt	25%	20%
	35	Arguments	35% *	20%
	36	Relationship problems	35% *	35% *
	37	Sleeping in bed	5%	5%
	38	Shame	20%	5%
	39	Sexuality	10%	15%
Health Concerns	40	Health concerns	0%	0%

Items marked with * indicate “problematic” items; meaning ≥30% of patients found it to be not relevant or comprehensible

We performed further exploratory analyses by matching responses to demographic characteristics. Women and older participants tended to rate more items as relevant. Women and patients who reported suffering from multiple diseases stressed the importance of the domains “Daily Activities” and “Social Interaction”, while patients who reported being encouraged by their partner to undergo sleep diagnostics tended to rate items from “Daily Activities”, “Memory and Concentration”, “Emotions”, and “Social Interaction” as largely irrelevant. Finally, all patients were asked whether anything was missing from the questionnaire. With the exception of the topic “quality of therapy,” no additional topics were identified as missing. All participants emphasized that the questionnaire was overall comprehensive and easy to understand, and that even if specific questions did not apply to them personally, they could apply to other affected individuals.

### Online expert survey

In total, *N* = 14 experts participated in an online survey sent by email in February 2026, who were selected by snowball sampling. The sample characteristics are presented in Table 4. For relevance, 14 items—predominantly from “Daily Activities”, “Emotions”, and​ “Social Interactions”—fell below the I‑CVI threshold of 0.78 (range: 0.43–1.00). REL D‑CVI values were heterogeneous, typically between 0.80 and 0.90 (range: 0.66–0.96), and the REL S‑CVI was 0.79. Especially sleep physicians rated the items for the domain “Daily Activities” as less relevant.

**Table 4:** Overview of characteristics of experts participating in an online survey

Variable	Categories	Absolute (%)
Sex	Male	7 (50%)
	Female	7 (50%)
Age group (years)	30–39	3 (21.43%)
	40–49	5 (35.71%)
	50–59	1 (7.14%)
	60–69	5 (35.71%)
Professional background	Sleep physician	7 (50%)
	Sleep scientist	4 (28.57%)
	Medical assistant	1 (7.14%)
	Other* (study coordinator, nurse)	2 (14.28%)
Experience in sleep medicine/science in years	1 – <2 years	1 (7.14%)
	2 – <6 years	4 (28.57%)
	6 – <10 years	2 (14.29%)
	≥ 10 years	7 (50%)
Ever used PROMs in professional context	Yes	12 (85.71%)
	No	0
	Unsure	2 (14.28%)

For comprehensibility, three items, mainly from “Daily Activities” and “Nocturnal Symptoms”, fell below the I‑CVI threshold of 0.78 (range: 0.71–1.00). COM D‑CVI values were consistently close to 0.90, with the lowest value in “Daily Activities” (range: 0.79–0.96). The COM S‑CVI was 0.89. For a detailed description of the CVI placements for relevance and comprehensibility see Table 5. Five experts recommended reducing the response scale to four or five categories, due to difficulties differentiating between options, e.g. “occasionally” and “rarely.” The recall period was predominantly judged as appropriate. Experts showed limited consensus regarding missing content, with almost no two experts mentioning the same aspects twice. Some examples were including the concept of tiredness into the domain sleepiness, amnestic aphasia into the domain of memory and concentration and early awakening in the domain of sleep quality. One exception that was mentioned multiple times was the recommendation to add “observed by a bed partner” to “Nocturnal Symptoms” items asking about snoring and choking at night.

**Table 5:** CVI per items and domain for relevance and comprehensibility of expert survey

Domain	Item	D-CVI REL	D-CVI COM	I-CVI REL	I-CVI COM
Nocturnal Symptoms	Snoring	0.84	0.90	1.00	1.00
	Nocturia			0.86	1.00
	Choking			0.86	0.79
	Restless sleep			0.71*	0.71*
	Dry mouth			0.79	1.00
	Headache			0.64*	0.93
Sleepiness	Fighting sleepiness	0.94	0.94	1.00	1.00
	Falling asleep			0.93	1.00
	Conversation			0.79	0.93
	Watching something			1.00	0.93
	Places			1.00	0.86
Tiredness	Feeling tired	0.83	0.86	0.86	0.79
	Energy			0.71*	0.86
	Waking up tired			0.93	0.93
Daily Activities	Most important activity	0.80	0.79	0.86	0.86
	Energy main activity			0.71*	0.79
	Performance			0.93	0.71*
	Hobbies			0.79	0.86
	Household			0.71*	0.71*
Unsafe Situations	Sleepiness	0.96	0.93	1.00	0.93
	Traffic concerns			0.93	0.93
Memory & Concentration	Forgetfulness	0.79	0.86	0.71*	0.79
	Concentration			0.86	0.93
Sleep Quality	Falling asleep	0.82	0.96	0.79	0.93
	Staying asleep			0.86	1.00
Emotions	Low mood	0.69	0.92	0.79	0.93
	Anxiety			0.50*	0.93
	Short temper			0.50*	0.79
	Irritability			0.79	1.00
	Daily life			0.71*	0.93
	Emotional reaction			0.86	0.93
Social Interactions	Snoring	0.66	0.89	0.71*	1.00
	Time with family			0.79	0.86
	Guilt			0.43*	0.79
	Arguments			0.43*	0.86
	Relationship problems			0.71*	1.00
	Sleeping in the same bed			0.79	0.93
	Shame			0.64*	0.93
	Sexuality			0.79	0.79
Health Concerns	Health concerns	0.86	0.93	0.86	0.93

Items marked with * did not meet the I-CVI threshold of 0,78 (applied to relevance and comprehensibility)

### Final revision

In total eight changes were made to the harmonized translated version for final revision. First, a short introduction of OSA was given due to patients repeatedly advising that OSA might be an unknown term for patients visiting the clinic for the first time to get tested. Second, the line “observed by bedpartner” was added to the items “snoring” and “choking” within the domain of “Nocturnal symptoms”, due to patients and experts consistently raising this as an important experience and aspect in clinical routine. Third, “painful” was cut from the item “dry mouth” within the domain of “Nocturnal symptoms” due to not being applicable to any patient. Fourth and fifth, short definitions of sleepiness and tiredness were added to the respective domain (“Sleepiness here refers to a physical urge to fall asleep”; “tiredness” here refers to a lack of energy and daytime exhaustion”) as patients consistently confused the terms and expert pointed at a lack of definition. Sixth, response categories and item wording were unified to the “never-always-scale within the domain “Daily Activities”, as most patients reported difficulties with switching response formats within the domain. Seventh, the response option “not applicable” was changed to “I do not drive a car/operate heavy machinery” within the domain of “unsafe situation”, due to most patients expressing difficulties differentiating between “not applicable” and “never” upon inquiry, sometimes confusing the meaning completely. The same was done to two questions within the domain “Social interactions” asking about problems in partnership and sleeping in the same bed with a partner, where “not applicable” and “not at all” was sometimes confused, therefore, we changed the category to “I do not have a partner”. Eighth, the phrase “due to sleep problems” was added to two items, assessing feelings of shame toward family and friends and problems in romantic relationships respectively within the domain “Social interactions”. Many patients reported uncertainty about how to answer these items because, in their original wording, they did not appear to relate to their sleep condition and the intended relevance was unclear. Participants therefore explicitly recommended adding this brief qualifier to clarify attribution.

## Discussion

This study presents the translation and cognitive validation the German version of the Patient-Reported Apnea Questionnaire, resulting in a linguistically and culturally sound instrument that preserves the meaning of the original (translated version in supplements). The translation process resolved minor wording differences while maintaining semantic equivalence. Furthermore, cognitive interviews with 20 patients and an expert survey with 14 experts demonstrated decent relevance, comprehensibility, and overall acceptability of the questionnaire, while adjustments were made to elevate comprehensibility and the flow of the questionnaire, like specifying items or harmonizing response options. Regarding the translation process, both the German and Dutch languages are closely related and belong to the same linguistic group [23], hence sentence structure and wording differed only slightly. Evidence from Dutch–German contrastive research suggests that, despite close relatedness, German shows a stronger tendency toward compact, nominal constructions (e.g., compounding) and Dutch more often using verb-based alternatives such as infinitive structures. This supports the rationale for our translation decisions, acknowledging language-specific conventions while preserving semantic equivalence [24].

Even though the questionnaire was perceived well, some items were prone to criticism or poor comprehensibility. Specifically items asking about daily activities and energy level regarding task, work and household were affected. This may reflect cross-cultural differences in how “daily activities” are conceptualized, with German respondents potentially adopting a more work-centred interpretation, whereas Dutch respondents may more readily integrate private and family-related responsibilities into the notion of everyday functioning and work–life balance [25], which may lead to an enhanced focus if pressure or imbalance occurs within these areas of life. It has to be stressed, however, that the PRAQ uses items form the MOSAS, QSA and SAQLI, which were developed in Italy and Canada (French language for QSQ and English language for SAQLI) respectively, thus picking from a culturally diverse item pool. Patients also expressed difficulty differentiating between the emotions of “having a short fuse”, feeling “irritated” and “having a strong emotional reaction to everyday situations”. This might be due to culturally different perceptions and expressions of emotions, yet more plausibly due to semantic understanding of these feelings and additional burden. Our evidence points towards emotional burden as an explanatory factor, since people with decreased HRQOL and high burden due to comorbidities gave distinct answers to each item (e.g. referring to “exploding” when asked about having a short fuse and in other situations feeling like crying regarding housework when asked about strong emotional response). This seems plausible considering the close connections between OSA and mental disorders such as anxiety and depression [26] and the fact that sleep loss and sleep fragmentation, as a regular occurrence in untreated OSA, are heavily linked to emotional dysregulation [27]. Furthermore, patient-reported burden and clinical risk are distinct dimensions of OSA. Patients with little perceived sleepiness or those referred to a clinic by their partners may still require diagnostic assessment and treatment based on clinical findings. The PRAQ is designed to assess HRQOL in patients with OSA, rather than to screen for OSA, quantify health-related risk, or assess readiness to seek care. Therefore, low relevance ratings for emotional or social items in our sample should not be interpreted as evidence that these domains are unimportant in the broader OSA population.

Sleep experts generally rated comprehensibility highly, whereas relevance was lower on average, leading to some items asking about daily activities, emotions and social interactions displaying insufficient S-CVI values. We realized that mainly items asking about similar aspects (such as energy with main daily activity and performance with main daily activity) are affected. This might correlate with the fact that to some experts stressed that the questionnaire is indeed comprehensive, yet to the expense of feasibility and completion time. In addition, some items were reassigned from their original domain to other domains. (e.g., aspects of sleep quality were sorted to the domain “Nocturnal Symptoms”), suggesting implications for the conceptual grouping of items to be tested in psychometric validation.

For the final revision of the PROM, eight modifications were implemented based on patient and expert feedback to improve comprehensibility; however, some concerns regarding content validity remain. Items falling below the predefined threshold were predominantly located within the functional and psychosocial domains, whereas symptom-related domains, including tiredness and sleepiness, demonstrated high relevance. This may reflect their close association with clinically established outcomes and greater awareness of these symptoms, particularly among clinicians. Nevertheless, for some patients, emotional distress or social impairment may represent the most substantial consequences of OSA. Previous studies have shown that patients in clinical trials often place greater emphasis on QOL outcomes, whereas clinicians may prioritize more traditional clinical endpoints, such as physical functioning and survival [28]. The PRAQ was developed to bring patient-reported problems into clinical consultations and to support outcome assessment [16]. Its use may help identify concerns that warrant discussion alongside clinical findings, yet measuring these concerns does not itself establish that they can be resolved by OSA treatment. Furthermore, although the PRAQ was well received by patients, clinicians perceived its usefulness as limited, which restricted its potential to enhance patient-centered care in clinical practice, as shown in a former study [29]. This framework helps explain why clinicians, such as sleep physicians in the online survey, give lower relevance ratings of QOL items that appear insufficiently actionable, poorly aligned with clinical workflows, or secondary to disease-focused outcomes, such as emotional coping or social burden in this case, which is (usually) not the primary focus of sleep physicians. While clinicians prioritise established clinical endpoints, QOL outcomes are potentially overlooked due to time constraints. The PRAQ may help identify patient priorities, although its clinical utility requires further evaluation, especially on aspects such as psychometric properties, cost-effectiveness, acceptability to patients and clinicians, and potential burden on and benefits for clinical consultations. Furthermore, concepts such as the WHO’s International Classification of Functioning, Disability and Health provides a framework for understanding health-related functioning across body functions, activities and participation, and environmental factors. Although the PRAQ did not cover all classification domains according to a review [30], the authors found it to have the second-highest percentage of ICF domain coverage among the OSA-specific PROMs evaluated, while only the SAQLI encompassed all.

## Limitation

The present work has several limitations. First, the generalizability of the findings is limited by context and sample characteristics. The presented item relevance and reported redundancy are likely subgroup-dependent due to a sample of predominantly retired and older people who have established OSA therapy, thus underrepresenting symptoms and activity constraints that are more salient pre-treatment or in working populations. Recall bias must be taken into account in this context. Further content-validity assessment in newly diagnosed and untreated patients would strengthen the evidence for the German version. Furthermore, as highlighted by Meadows [31], recommendations on sample size in cognitive interviews vary considerably, ranging from 6 to 10 interviews in classical guidance to 22 up to 90 participants in more recent publications. Although our sample of 20 patients adhered to the study protocol and is in line with established guidelines, we cannot exclude the possibility that additional interviews might have identified further issues. Second, we did not use a transcript to analyse the patient interviews, potentially missing subtle implications and important content, which poses a methodological limitation. Third, Patients contributed through cognitive interviews, whereas experts provided structured item ratings. The absence of quantitative patient ratings limited direct comparison between the two perspectives. Qualitative interviewing can identify reasons for interpretation difficulties, but concerns may remain undetected if they are not elicited through targeted probing. Future assessment of content validity should combine cognitive interviewing with structured patient ratings and explicit questions about missing concepts. Comprehensiveness should be assessed for the instrument as a whole, in addition to item-level relevance and comprehensibility. Fourth, the response processes may have influenced item-level conclusions. The overlap between “not relevant” and “not comprehensible” suggests that respondents may not have consistently distinguished these constructs in some cases, where even after an explanation of the concept patients deemed an item as not relevant due to poor understanding. Fifth, some final modifications may affect construct fidelity. Because the PROM targets patient-perceived HRQOL, qualifiers such as “observed by a bed partner” could shift the focus toward external observation rather than self-experienced burden, which could be seen as a restriction. We still decided to include this sentence because of the frequent recommendation by both patients and experts and the fact that reporting about a second-hand experience about one’s own health can still be deemed as patient-perceived. Sixth, the response option design entails a precision–usability trade-off. More response categories, as in the original version, may increase sensitivity but can reduce usability when adjacent options are difficult to discriminate, as mentioned by some experts and a few patients, yet alternative scaling should be evaluated empirically, thus we kept the 7-point scale. Seventh, the modifications made to the final version may endanger comparability with the original PROM. While we kept the number of items, some additions to items and the adjustment to the response option in “Daily Activities” domain may necessitate additional validation. Eighth, the adaptation targeted standard written German. Comprehensibility in the study sample does not establish equivalence across regional dialects or other German-speaking healthcare settings. Further evaluation may be required before use in Austria or Switzerland.

## Conclusion

Overall, the German version of the PRAQ demonstrates decent content validity and comprehensibility, although some items may be less relevant for a subset of patients. Conversely, items considered less relevant overall were highly important for specific patient groups with greater disease burden, reflecting the heterogeneity of OSA-related experiences. As a comprehensive PROM, the PRAQ aims to capture a broad range of patient perspectives, which may not be equally relevant across all individuals. No essential aspects were identified as missing. The final version will serve as the basis for ongoing psychometric validation in German-speaking patients with OSA, including evaluation of structural validity, reliability, and responsiveness.

## Supplementary Information

Below is the link to the electronic supplementary material.

Supplementary Material 1

Supplementary Material 2

## Data Availability

The datasets used during the current study are available from the corresponding author on reasonable request.
