# Supplementary material for: Forward-backward translation and content validity study of the German version of the Patient-Reported Apnea Questionnaire
Source: J Patient Rep Outcomes. 2026 Nov 5;10:204. doi: 10.1186/s41687-026-01246-3 (PMC13743691; doi:10.1186/s41687-026-01246-3)
Supplement: Supplementary file 1 [file 41687_2026_1246_MOESM1_ESM.pdf]

PRAQ-D

Dieser Fragebogen bezieht sich auf die Beschwerden, die möglicherweise mit einer Schlafapnoe zu tun haben. Schlafapnoe bedeutet, dass es im Schlaf wiederholt zu Atempausen kommt, wodurch der Schlaf gestört wird und Folgen für die Gesundheit auftreten können

Die Fragen beziehen sich auf die letzten vier Wochen.

Nächtliche Symptome

Hatten Sie in den letzten 4 Wochen Probleme mit:

	überhaupt kein Problem	geringes Problem	geringes bis mäßiges Problem	mäßiges Problem	mäßiges bis großes Problem	großes Problem	sehr großes Problem
lautem Schnarchen? (ggf. durch Bettpartner*in bemerkt)	O	O	O	O	O	O	O
häufigem Aufwachen, um Wasser zu lassen?	O	O	O	O	O	O	O
aufwachen in der Nacht mit dem Gefühl, zu ersticken? (ggf. durch Bettpartner*in bemerkt)	O	O	O	O	O	O	O
dem Gefühl, unruhig zu schlafen?	O	O	O	O	O	O	O
einem trockenen Mund beim Aufwachen?	O	O	O	O	O	O	O
morgendliche Kopfschmerzen beim Aufwachen?	O	O	O	O	O	O	O

Schläfrigkeit

(Schläfrigkeit meint hier einen körperlichen Drang einzuschlafen)

Hatten Sie in den letzten 4 Wochen ein Problem mit folgenden Symptomen:

	überhaupt kein Problem	geringes Problem	geringes bis mäßiges Problem	mäßiges Problem	mäßiges bis großes Problem	großes Problem	sehr großes Problem
Tagsüber gegen die Müdigkeit ankämpfen müssen?	O	O	O	O	O	O	O
Plötzliches Einschlafen?	O	O	O	O	O	O	O
Schwierigkeiten, während eines Gesprächs wach zu bleiben?	O	O	O	O	O	O	O
Schwierigkeiten, wach zu bleiben, während man sich etwas angesehen hat? (Konzert, Film, Fernsehsendung)?	O	O	O	O	O	O	O
Einschlafen zu Zeiten oder an Orten, an denen es nicht beabsichtigt ist?	O	O	O	O	O	O	O

Müdigkeit

(Müdigkeit meint hier einen Energiemangel und Erschöpfung am Tag)

Hatten Sie in den letzten 4 Wochen ein Problem mit folgenden Symptomen:

	überhaupt kein Problem	geringes Problem	geringes bis mäßiges Problem	mäßiges Problem	mäßiges bis großes Problem	großes Problem	sehr großes Problem
Starker Müdigkeit?	O	O	O	O	O	O	O
Einem Mangel an Energie?	O	O	O	O	O	O	O
Morgendliches Aufwachen mit dem Gefühl, noch immer müde zu sein?	O	O	O	O	O	O	O

Tägliche Aktivitäten

in den letzten 4 Wochen…:

	nie	selten	ab und zu	regelmäßig	oft	sehr oft	immer
wie oft hatten Sie Mühe, Ihre wichtigste tägliche Tätigkeit auszuführen (z. B. Arbeit, Studium, Kinderbetreuung, Haushalt)?	O	O	O	O	O	O	O
wie oft hat Ihre gesamte Energie nur für Ihre wichtigste tägliche Tätigkeit gereicht (zum Beispiel Arbeit, Studium, Kinderbetreuung, Haushalt)?	O	O	O	O	O	O	O

	nie	selten	ab und zu	regelmäßig	oft	sehr oft	immer
wie oft hatten Sie das Gefühl bei Ihrer wichtigsten Tätigkeit weniger leistungsfähig zu sein (zum Beispiel Arbeit, Studium, Kinderbetreuung, Haushalt)?	O	O	O	O	O	O	O
wie oft hatten Sie Mühe, Energie für Ihre Hobbys zu finden?	O	O	O	O	O	O	O
wie viel Mühe hat es Sie gekostet, Aufgaben zuhause und im Haushalt zu erledigen?	O	O	O	O	O	O	O

Unsichere Situationen

in den letzten 4 Wochen…:

	Ich fahre kein Auto/ bediene keine Maschinen	überhaupt nicht	ein bisschen	ein bisschen bis mäßig	mäßig	mäßig bis stark	stark	sehr stark
hatten Sie Probleme beim Autofahren aufgrund von Schläfrigkeit?	O	O	O	O	O	O	O	O
haben Sie sich wegen Ihrer Schläfrigkeit Sorgen um Ihre eigene Sicherheit oder die Sicherheit anderer gemacht (z. B. im Straßenverkehr oder beim Bedienen von Maschinen)?	O	O	O	O	O	O	O	O

Gedächtnis und Konzentration

in den letzten 4 Wochen…:

	nie	selten	ab und zu	regelmäßig	oft	sehr oft	immer
waren Sie manchmal vergesslich?	O	O	O	O	O	O	O
hatten Sie manchmal Mühe sich zu konzentrieren?	O	O	O	O	O	O	O

Schlafqualität

Hatten Sie in den letzten 4 Wochen ein Problem mit folgenden Symptomen?

	überhaupt kein Problem	geringes Problem	geringes bis mäßiges Problem	mäßiges Problem	mäßiges bis großes Problem	großes Problem	sehr großes Problem
Einschlafen, wenn Sie abends ins Bett gehen?	O	O	O	O	O	O	O
Wiedereinschlafen, nachdem Sie nachts aufgewacht sind?	O	O	O	O	O	O	O

Emotionen

in den letzten 4 Wochen…:

	nie	selten	ab und zu	regelmäßig	oft	sehr oft	immer
wie oft fühlten Sie sich niedergeschlagen oder hoffnungslos?	O	O	O	O	O	O	O
wie oft fühlten Sie sich ängstlich?	O	O	O	O	O	O	O
wie oft hatten Sie ein „kurze Zündschnur“?	O	O	O	O	O	O	O
wie oft hatten Sie das Gefühl, dass Sie Ihren Alltag nicht bewältigen konnten?	O	O	O	O	O	O	O
wie oft fühlten Sie sich gereizt?	O	O	O	O	O	O	O
wie oft hatten Sie eine starke emotionale Reaktion auf alltägliche Dinge?	O	O	O	O	O	O	O

Soziale Interaktionen

in den letzten 4 Wochen…:

	überhaupt nicht	ein bisschen	ein bisschen bis mäßig	mäßig	mäßig bis stark	stark	sehr stark
haben Sie sich schon einmal unwohl gefühlt, weil andere Ihr Schnarchen störend fanden?	O	O	O	O	O	O	O
war es für Sie ein Problem, dass Sie manchmal keine Energie oder Lust hatten,	O	O	O	O	O	O	O

etwas mit Ihrer Familie oder Ihren Freunden zu unternehmen?

	überhaupt nicht	ein bisschen	ein bisschen bis mäßig	mäßig	mäßig bis stark	stark	sehr stark
hatten Sie Schuldgefühle gegenüber Ihrer Familie oder Ihren Freunden wegen Ihrer Schlafprobleme?	O	O	O	O	O	O	O
haben Sie sich belastet gefühlt, weil Sie viel gestritten haben?	O	O	O	O	O	O	O

	ich habe keinen Partner/ keine Partnerin	überhaupt nicht	ein bisschen	ein bisschen bis mäßig	mäßig	mäßig bis stark	stark	sehr stark
hatten Sie schon mal Probleme in Ihrer Beziehung zu Ihrem Partner wegen Ihrer Schlafprobleme?	O	O	O	O	O	O	O	O
hat es Sie gestört, dass Sie (vielleicht) nicht mit Ihrem Partner im selben Zimmer schlafen konnten?	O	O	O	O	O	O	O	O

	überhaupt nicht	ein bisschen	ein bisschen bis mäßig	mäßig	mäßig bis stark	stark	sehr stark
haben Sie sich gegenüber anderen für Ihre Schlafapnoe oder deren Behandlung geschämt?	O	O	O	O	O	O	O

	keine Antwort	überhaupt nicht	ein bisschen	ein bisschen bis mäßig	mäßig	mäßig bis stark	stark	sehr stark
hatten Sie ein Problem mit unbefriedigender und/oder geringer sexueller Aktivität (allein oder mit einem/einer Partner*in)?	O	O	O	O	O	O	O	O

Gesundheitliche Sorgen

in den letzten 4 Wochen…:

	überhaupt nicht	ein bisschen	ein bisschen bis mäßig	mäßig	mäßig bis stark	stark	sehr stark
haben Sie sich Sorgen über andere Erkrankungen gemacht, die mit Schlafapnoe in Zusammenhang stehen könnten? (z. B. Diabetes, Bluthochdruck, Herz-Kreislauf-Erkrankungen, Übergewicht)?	O	O	O	O	O	O	O
