# Supplementary material for: Forward-backward translation and content validity study of the German version of the Patient-Reported Apnea Questionnaire
Source: J Patient Rep Outcomes. 2026 Nov 5;10:204. doi: 10.1186/s41687-026-01246-3 (PMC13743691; doi:10.1186/s41687-026-01246-3)
Supplement: Supplementary file 2 [file 41687_2026_1246_MOESM2_ESM.docx]

**ISPOR Checklist for Translation and Cultural Adaptation**

| **Step** | **Description** | **Comment** | **Page** |
| --- | --- | --- | --- |
| Preparation | Obtain permission to use instrument |  | p. 5 line 91-92 |
|  | Invite instrument developer to be involved |  | p. 6 line 112 |
|  | Develop explanation of concepts in instrument |  | p. 5 line 92-93 |
|  | Recruit key in-country persons to the project | No single “key person” was designated for each language; instead, all translators were considered equally important and played an integral role throughout the entire process. The risks for not doing this are rated as “not applicable” by ISPOR. | NA |
| Forward translation | Development of at least two independent forward translations |  | p. 5 line 94-95 |
|  | Provision of explanation of concepts in the instrument to the key in-country persons and forward translators |  | p. 5 line 99-100 |
| Reconciliation | Reconciliation of the forward translations into a single forward translation |  | p. 5 line 103-105 |
| Back translation | Back translation of the reconciled translation into the source language |  | p. 5/6 line 105-109 |
| Back translation review | Review of the back translations against the source language |  | p. 6 line 109-111 |
| Harmonization | Harmonization of all new translations with each other and the source version |  | p. 6 line 109-113 |
| Cognitive debriefing | Cognitive debriefing of the new translation, usually with patients drawn from the target population |  | p. 6/7 line 115-143 |
| Review and finalization | Cognitive debriefing results are reviewed and the translation finalized |  | p. 6 line 111-112 |
| Proofreading | The finalized translation is proofread | Completed, but this was not explicitly stated in the original version and has now been added. | p. 6 line 111-112 |
| Final report | Report is written on the development of the translation |  | This paper, specifically p. 5-6 line 91-164 |

**Reference:** Wild, D., Grove, A., Martin, M., Eremenco, S., McElroy, S., Verjee-Lorenz, A., Erikson, P., & ISPOR Task Force for Translation and Cultural Adaptation (2005). Principles of Good Practice for the Translation and Cultural Adaptation Process for Patient-Reported Outcomes (PRO) Measures: report of the ISPOR Task Force for Translation and Cultural Adaptation. *Value in health : the journal of the International Society for Pharmacoeconomics and Outcomes Research*, *8*(2), 94–104. <https://doi.org/10.1111/j.1524-4733.2005.04054.x>
